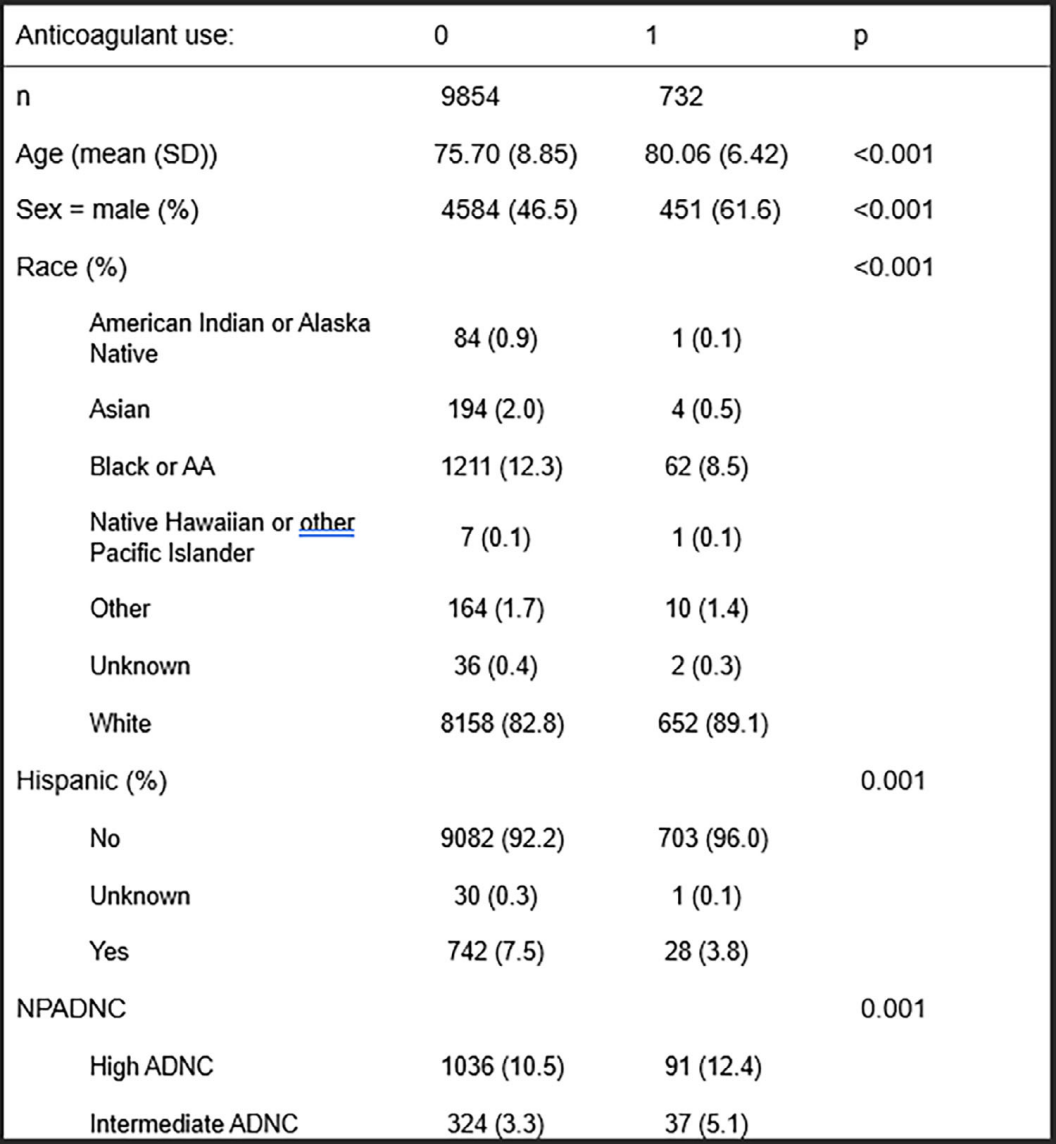# Anticoagulation as a Limitation to New Alzheimer's Disease Anti‐Amyloid Monoclonal Antibodies therapy

**DOI:** 10.1002/alz70860_102086

**Published:** 2025-12-23

**Authors:** Zafarullah M Chaudhary, Tovia Jacobs, Matin Soeizi, Ricardo S. Osorio, Sakina Ouedraogo Tall

**Affiliations:** ^1^ NYU Grossman School of Medicine, New York, NY, USA; ^2^ New York City Health and Hospitals, New York, NY, USA; ^3^ NYU Alzheimer's Disease Research Center, New York, NY, USA

## Abstract

**Background:**

Over 6 million Americans are living with Alzheimer's disease (AD). AD poses a significant public health challenge. Following newly approved Anti‐Amyloid Monoclonal Antibodies, such as lecanemab (Leqembi), for the treatment of AD at Mild Cognitive Impairment (MCI) and mild dementia stages, protocols were created by institutions for their safe use given concerns for Amyloid‐Related Cerebral Imaging Abnormalities (ARIA). People on anticoagulation therapy are excluded from treatment due to increased risk for ARIA. Atrial Fibrillation (AFib) affects 9% of individuals 65 and older and anticoagulation is the standard of care for stroke prevention. This study examines the prevalence of anticoagulant use in individuals with AD at MCI and mild dementia stages.

**Method:**

Using the National Alzheimer's Coordinating Center (NACC) database, we retrospectively reviewed data from individuals with a clinical diagnosis of AD at MCI and mild dementia stages, and a MMSE of 20 or above. Anticoagulant use and their indications were recorded. We further examined those with available neuropathology data. Statistical analyses were conducted using R software, Pearson's chi‐squared test for categorical variables and t‐tests or one‐way analysis of variance (ANOVA) for continuous variables.

**Result:**

Of 10,586 individuals with a clinical diagnosis of AD at MCI (*n* = 6,211) or mild dementia stage (*n* = 4,375), 732 (6.9%) were on anticoagulant. Participants on anticoagulant were older (mean age 80.06 vs. 75.70, *p* <0.001), more likely to be male (61.6% vs. 46.5%, *p* <0.001), and White individuals (89.1% vs. 82.8%, *p* <0.001). Warfarin was the most common anticoagulant (94.8%), followed by rivaroxaban (3.6%) and apixaban (1.6%). Anticoagulant users had higher rates of AFib (53.0% vs. 3.2%, *p* <0.001). Amongst those with AD neuropathologic changes (*n* = 1,671), anticoagulation therapy was recorded in 8.56 %. No AD pathology were found in 128 presumed cases of AD, 14% of those were on anticoagulant.

**Conclusion:**

Our study reveals that a large number of individuals with AD are on anticoagulation therapy for AFib. Considering the high prevalence of AFib as a cause of anticoagulation therapy, treatment protocols for AFib should prioritize an individualized approach and include early detection of AD. Future AD clinical trials should focus on expanding treatment access for those on anticoagulant while ensuring safety.